# Gamma-induced interconnected networks in microporous activated carbons from palm petiole under NaNO_3_ oxidizing environment towards high-performance electric double layer capacitors (EDLCs)

**DOI:** 10.1038/s41598-023-40176-8

**Published:** 2023-08-09

**Authors:** Nurulsafeelanaria Benwannamas, Tanagorn Sangtawesin, Murat Yilmaz, Kotchaphan Kanjana

**Affiliations:** 1 Department of Chemistry, School of Science, Walailak University, Tha Sala, Nakhon Si Thammarat 80160 Thailand; 2 Functional Materials and Nanotechnology Center of Excellence, Walailak University, Tha Sala, Nakhon Si Thammarat 80160 Thailand; 3 Thailand Institute of Nuclear Technology, Ongkharak, Nakhon Nayok 26120 Thailand; 4 Department of Chemistry and Chemical Processing Technologies, Bahçe Vocational School, Osmaniye Korkut Ata University, 80000 Osmaniye, Turkey

**Keywords:** Energy science and technology, Materials science

## Abstract

Activated carbons (ACs) were developed from palm petiole via a new eco-friendly method composed of highly diluted H_2_SO_4_ hydrothermal carbonization and low-concentration KOH-activating pyrolysis followed by gamma-induced surface modification under NaNO_3_ oxidizing environment. The prepared graphitic carbons were subsequently used as an active material for supercapacitor electrodes. The physiochemical properties of the ACs were characterized using field emission scanning electron microscope–energy dispersive X-ray spectroscopy, N_2_ adsorption/desorption isotherms with Brunauer–Emmett–Teller surface area analysis, Fourier transform infrared spectroscopy, X-ray diffraction and Raman spectroscopy. The electrochemical performance of the fabricated electrodes was investigated by cyclic voltammetry, galvanostatic charge–discharge and electrochemical impedance spectroscopy. Even treated with extremely low H_2_SO_4_ concentration and small KOH:hydrochar ratio, the maximum S_BET_ of 1365 m^2^ g^−1^ for an AC was obtained after gamma irradiation. This was attributed to radiation-induced interconnected network formation generating micropores within the material structure. The supercapacitor electrodes exhibited electric double-layer capacitance giving the highest specific capacitance of 309 F g^−1^ as well as excellent cycle stability within 10,000 cycles. The promising results strongly ensure high possibility of the eco-friendly method application in supercapacitor material production.

## Introduction

Recently fossil energy becomes less attractive due to its limited long-term energy supply and environmental issues. On the other hand, alternative energy has been increasingly taken into consideration to ensure sustainability and clean environment. Regardless of energy sources, all renewable energy processing requires a stable and reliable energy storage system (ESS). Considerable attentions have been drawn to supercapacitors as they offer high stability, rapid charging ability and wide operating temperature. This class of energy storage devices also exhibits high power density with energy efficiency up to 98%^1^. Unlike traditional solid dielectric capacitors, the total capacitance of supercapacitors relies essentially on electric double-layer capacitance (EDLC) and electrochemical pseudocapacitance. In fact, the capacitance value of a supercapacitor is influenced by several factors, namely specific surface area, pore structure, electrical conductivity and surface functionality of the electrodes. EDLCs normally provide specific surface area greater than 500 m^2^ g^−1^ leading to much higher specific capacitance than those of conventional capacitors. Fast charge accumulation reversibility in EDLCs also allows completion of charge/discharge cycle within seconds^2^. As opposed to rechargeable batteries whose cyclic life is shorten after a number of chemical reaction-based charge/discharge processes, EDLCs can maintain exceptional electrode structure and high capacity even after millions of operating cycles^3^. Unfortunately EDLCs commercially available still suffer from a much lower energy density (< 10 Wh kg^−1^) when compared to those of batteries (35–40 Wh kg^−1^)^4^. In fact, the energy density and total capacitance of a supercapacitor can be improved when introducing an additional charge storage–charge transfer mechanism so-called pseudocapacitance to the electrode material. The mechanism takes place through fast and reversible oxidation–reduction reactions at the electrode/electrolyte interface and in the bulk electrode material. In other words, this faradaic process helps decrease the energy gap between EDLCs and batteries^5,6^. Pseudocapacitive behaviors in SCs are mainly governed by the presence of heteroatom-containing functional groups on the electrode surface. Metal oxides, metal nitrides and conducting polymers are the most widely used materials in pseudocapacitor electrodes. The production process however requires complex procedures in combination with a number of toxic substances, thus posing environmental risks^5^.

Recently biomass-derived activated carbons (ACs) are considered an attractive choice for electrode active materials as this group of porous materials can be produced from low-cost and renewable agricultural wastes. They have been synthesized from a wide variety of starting materials, including potato, acorn shells, pistachio shells, peanut shells, algarroba wood, macadamia nut shells, walnut shells, corncob, durian shell, rubber seed shell and palm petiole^7–15^. Among alternatives, palm petiole (PP), produced during harvesting process of oil palm (*elaeis guineensis*), is material of interest since PP is largely generated from oil palm plantations, which are grown in Africa, South America, and Southeast Asia covering approximately 20 million hectares. Especially in Thailand, around 10.5 million tons of palm petioles are generated yearly^16^. More importantly, it has been shown that PP, containing 42.7% cellulose, 34.0% hemicellulose and 22.9% lignin, had porous nature even before treating^13^. To improve the energy storage performance of the electrode material, even a naturally porous AC precursor like PP still needs further activations. Generally carbon activation can be classified into two categories: (1) chemical activation via a high-temperature treatment of the precursor mixed with activating agents such as H_2_SO_4_, KOH, ZnCl_2_, H_2_O, H_3_PO_4_ and NaOH, and (2) physical activation through pyrolysis at high temperatures under activating gas such as CO_2_, steam, or gas mixture^10,12,17–22^. For chemical activation, low-cost H_2_SO_4_ is known to be a proper activating agent when it is used during hydrothermal carbonization of a biomass. During hydrothermal carbonization, this acidic agent can initially break large lignocellulosic structures into smaller parts and further promote polymerization of the small molecules first produced even under mild conditions. This series of reactions will give sulfonated biochar as a product^23^. KOH is another well-proven activating reagent which is suitable for pyrolysis at high temperature. When mixed with an AC precursor and further pyrolyzed, KOH can react with the sample generating a number of gaseous products creating porosity throughout the structure. The resultant ACs normally show a high specific surface area with mesopores^17,24^. The pore sizes of the synthesized ACs are comparable to that of K^+^ ion; therefore, this is extremely beneficial when the prepared electrode used with KOH electrolyte. After an activation or a series of activations, the resultant materials known as ACs are expected to have higher specific surface area and porosity which can facilitate the supercapacitor behaviors. Besides, a subsequent modification by radiation processing such as gamma irradiation can be introduced. Radiation processing is one of promising methods for modification of carbon materials since the approach is environmentally friendly and it offers possible large-scale production with uniformity. When a medium is gamma irradiated, a number of highly reactive chemical species so-called radiolytic primary species are generated and this medium is said to undergo gamma radiolysis. For example, gamma radiolysis of water produces (e)^−^_aq_, ^·^OH, H_2_O_2_, H^·^, H_2_ and HO_2_/O_2_^−^^25^. Material modification by gamma radiolysis is based on the chemical reactions between the radiolytic primary species and the material surface. The studies on carbon cloth demonstrated that upon gamma irradiation, surface phenolic groups and acidity of the carbon material were clearly increased when treated in air^26^, and the number of oxygenated groups on the sample surface was reduced in an alkaline medium^27^. Another publication regarding surface chemistry modification of activated carbon by gamma radiation also showed that desirable surface functionality and significantly increased sp^2^ hybridization were found in the post-irradiated materials^28^. It is therefore strongly evident that gamma irradiation on carbon structures can improve quantity of oxygenated functional groups on the material surface as well as its sp^2^ hybridization both of which can aid in facilitating charge-storage and charge delivery in a carbon-based supercapacitor. In addition, application of radiation processing can help reduce the use of hash chemicals and energy during the activation processes.

In this work, we propose an environmentally friendly method for palm petiole-derived AC production based on chemical and physical activations in conjunction with radiation processing. In spite of various studies on AC preparation from PP, to the best of our knowledge, there is no publication on PP activated carbon preparation via low concentration H_2_SO_4_/KOH chemical and physical activations in combination with gamma irradiation specifically for the purpose of supercapacitor application. The novelty of this research is to produce ACs from abundantly local palm petiole via a new environmentally friendly three-step strategy consisted of H_2_SO_4_ hydrothermal carbonization at 160 °C, KOH-activating pyrolysis under Ar atmosphere at the target temperatures of 700 and 800 °C, and gamma radiation treatment at different doses under NaNO_3_ oxidizing environment. This is also the first study to clearly demonstrate that gamma irradiation can aid in increasing the specific surface area of a carbon material by inducing interconnected networks of the micropores within the structure. The textural and chemical properties of the prepared activated carbons were characterized using various techniques. The materials were finally applied as an active material for a supercapacitor electrode, and its electrochemical behaviors were tested according to CV, GCD and EIS measurements.

## Material and method

### Material

Palm petiole (PP), biomass from abundantly available palm oil tree in Tha Sala district, Nakhon Si Thammarat, Thailand, was used as carbon precursor. At first, the petiole was sliced into thin and small pieces and dried at 90 °C for 15 h. Then, it was ground into powder with a mixer mill and a ball mill, respectively. Only the powder particles with the size up to 250 µm were collected for further steps. All the experiments were thoroughly conducted using deionized water (DI water).The chemicals including 98% sulfuric acid (H_2_SO_4_, RCI Labscan), 85% potassium hydroxide (KOH, KemAus), 37% hydrochloric acid (HCl, QReC) and 99% sodium nitrate (NaNO_3_, Loba Chemie) were all analytical grade and used as received.

### Preparation of activated carbons

For step 1, hydrothermal carbonization, H_2_SO_4_ was used as a catalyst for hydrolysis and cleavage of the biomass. Initially, 10 g of the biomass powder was dispersed in 100 mL 0.6%wt H_2_SO_4_ aqueous solution and stirred for 30 min. The mixture was then transferred into a stainless steel reactor with Teflon liner and cured at 160 °C for 12 h. After cooling down to room temperature, the obtained brown product, called hydrochar, was washed several times with DI water until pH reached neutral, filtered, and dried at 80 °C for 8 h. This washing step was done to eliminate organic by-products and to adjust pH as the pH value during hydrothermal carbonization normally decreased due to the formation of organic acids. Before further activation, the collected product was characterized with FTIR. For step 2, subsequently the hydrochar was mixed homogeneously with KOH (impregnation ratio of 1:1) and carbonized and activated via pyrolysis in the horizontal furnace tube at 700 and 800 °C for 2 h with the ramp rate of 10 °C/min under Ar atmosphere (flow rate of 100 ml/min). KOH was used as activator at this step to increase porosity. The resultant black products, known as activated carbons (ACs), were later dispersed in 10% HCl solution for 1 h and washed several times with DI water to remove the leftover ash contents and K^+^ ions on the material surface until neutral pH was achieved. HCl washing method can reduce ash content down to the range of 0–9.4%^29–31^.The solid products were later filtered and dried at 80 °C for 8 h. The as-prepared ACs at 700 and 800 °C were denoted as PP700 and PP800, respectively.

### Gamma irradiation

For step 3, gamma irradiation, 2 g of each activated carbon was dispersed in a 0.1 M NaNO_3_ solution making the total volume of 50 ml, and purged with N_2_ for 20 min. After purging, the mixture was irradiated with gamma ray from a cobalt-60 irradiator (30366 Ci, dose rate of 4 kGy/h) at the Thailand Institute of Nuclear Technology. The irradiation periods were 6.25, 12.5 and 25 h for the total doses of 25, 50, and 100 kGy, respectively. Harwell Amber Perspex dosimeter was used for dosimetry. The obtained products were washed with deionized water, filtrated, and dried overnight at 80 °C. The irradiated activated carbons were denoted as PP700_25, PP700_50, PP700_100, PP800_25, PP800_50 and PP800_100 where the digits following low dash represented the gamma dose. Besides, the activated carbons without irradiation (controlled set) were also used for comparison, and denoted as PP700_0 and PP800_0.

### Physicochemical characterization

Field emission scanning electron microscope–energy dispersive X-ray spectroscopy (FESEM–EDS) analysis was conducted to investigate the morphology and elemental composition of the materials (ZEISS, MERLIN Compact-Oxford, Aztec ED). The Brunauer–Emmett–Teller (BET) surface areas of the materials were obtained from ASAP2460 (at 300 °C degassing temperature)**.** T-plot and density functional theory (DFT) methods were also applied for porosity analysis. The variation of functional groups of hydrochar and activated carbons was investigated using fourier transform infrared spectroscopy (FTIR, BRUKERS, Tensor 27) with a scan range of 4000–500 cm^−1^. The crystallinity and chemical states of the samples were determined by powder X-ray diffraction (XRD, PANalytical) with a Cu Kα radiation, λ = 1.54056 Å and Raman spectroscopy (XploRA PLUS Raman, HARIBA) at 532 nm.

### Electrochemical measurement

To fabricate the supercapacitor electrode, 14 mg of activated carbon, 2.5 mg of a carbon black conductive additive, 10 µl of a polytetrafluoroethylene (PTFE) binder and 600 µl of ethanol were mixed. It should be noted that carbon black was used as a conductive additive since it has a better stacking structure compared to activated carbon, thus facilitating electron flow in the fabricated electrodes. The mixture was then sonicated for 30 min. The slurry was coated onto a graphite sheet with 1.5 cm × 2 cm active surface area. The loading mass of the active material was around 3–5 mg. The electrochemical tests were conducted using a potentiostat (Metrohm Autolab, PGSTAT302 N) with a three-electrode system in 1 M KOH electrolyte under ambient condition. Cyclic voltammetry (CV) curves were recorded at different scan rates (0.01–0.1 V/s) to investigate the electrochemical behaviors of the materials. Galvanostatic charge–discharge (GCD) was performed at varied current densities (1–10 A g^−1^) to study the charge–discharge nature of the prepared electrodes and to calculate the specific capacitance. Electrochemical impedance spectroscopy (EIS) in the frequency range of 100 kHz–0.1 Hz with 0.1 V was also recorded to detect the electrical response at the electrode interface.

## Results and discussion

**Figure 1:**
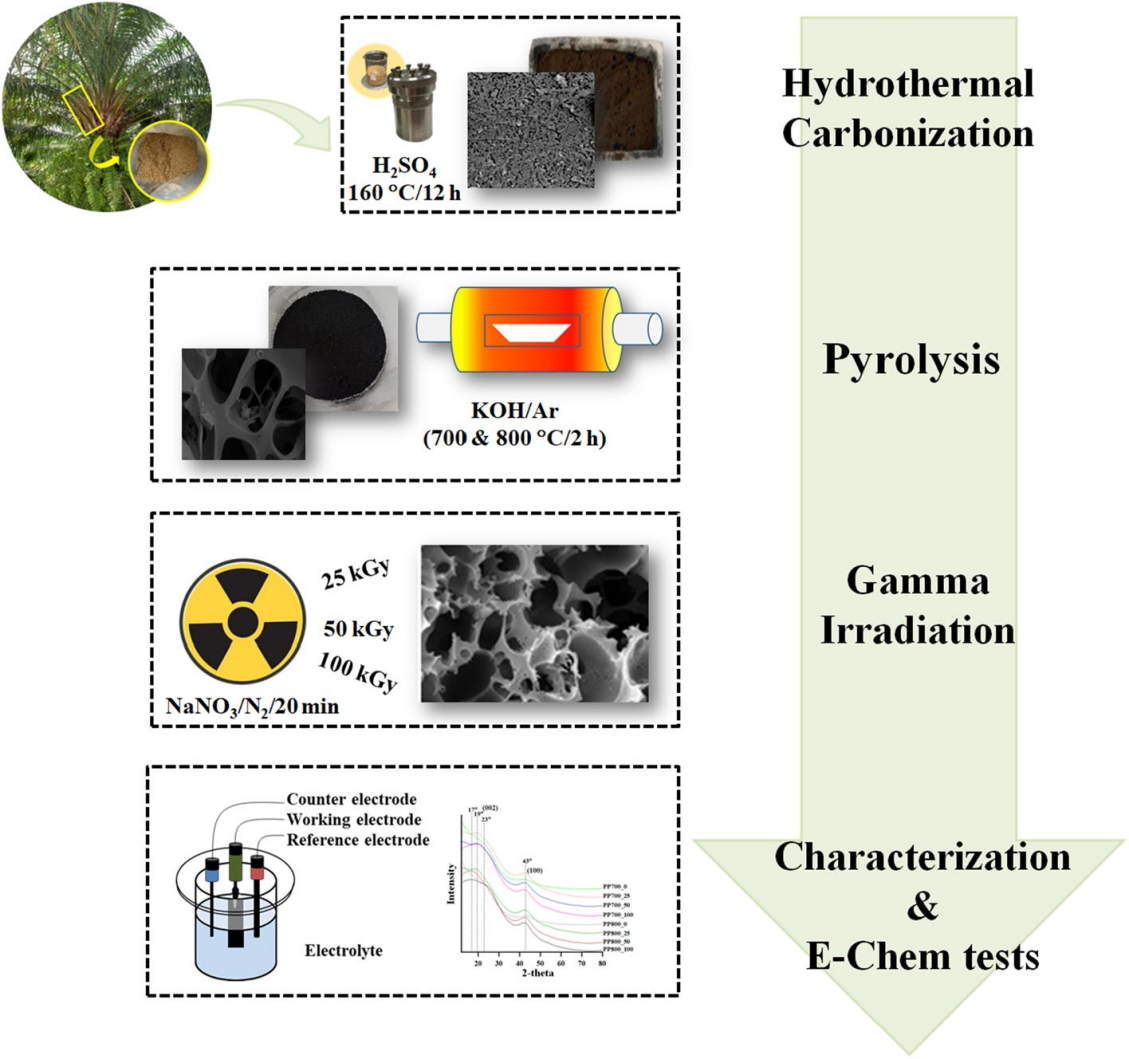
Experimental scheme.

In this study PP biomass was first hydro-thermalized with H_2_SO_4_ at 160 °C for 12 h. The hydrochar produced was later mixed homogeneously with KOH activating agent at the hydrochar:KOH ratio of 1:1. Subsequently the mixture was carbonized and activated through pyrolysis under Ar atmosphere at 700 and 800 °C for 2 h. The activated carbons obtained were then irradiated with gamma radiation in deaerated aqueous NaNO_3_ at the total doses of 25, 50 and 100 kGy. Subsequently, the post-irradiated products were characterized using FESEM-EDS, N_2_ adsorption/desorption isotherms with BET surface area analysis, FTIR, XRD and Raman spectroscopy. The porous carbons were finally used as supercapacitor electrode material and undergone electrochemical measurements including CV, GCD and EIS. The experimental scheme is described in Fig. 1. The yields of the activated carbons produced at 700 °C and 800 °C were 29.42 and 28.68%, respectively. The values were obtained from $\frac{{{\text{AC }}\,{\text{mass}} \times { }100}}{{{\text{mass }}\,{\text{of }}\,{\text{starting}}\,{\text{ material}}}}$.

In general, upon gamma irradiation water molecules are excited and ionized giving a number of highly reactive radicals and molecules called radiolytic primary species as shown in Reaction 1 (R1)^32^. The G-values, defined as the number of the species produced per 100 eV of energy absorbed, for (e^−^)_aq_, ^·^OH, H_2_O_2_, H^·^, H_2_, and HO_2_/O_2_^−^ are 2.70, 2.87, 0.61, 0.61, 0.43 and 0.026, respectively^25^. In the presence of NaNO_3_, the hydrated electrons (e^−^_(aq)_) and ^·^H produced react with NO_3_^-^ ions as described in Reaction 2 (R2) with the rate constant of 9.7 × 10^9^ M^-1^ s^-1^ and Reaction 3 (R3) with the rate constant of 1.0 × 10^7^ M^−1^ s^−1^, respectively^33–35^. R2 and R3 lead to e^-^_(aq)_ and ^·^H scavenging which also reduce the chain reactions generating H_2_^36^. As a result, the key radiolytic primary species remain in the medium are ^·^OH and H_2_O_2_, and the overall chemistry system is converted into a fully oxidizing condition. In a presence of activated carbon the remaining oxidizing species can therefore react with the surface functional groups leading to surface modification of the carbon materials.

R1 $${\text{H}}_{{2}} {\text{O}}\mathop{\longrightarrow}\limits^{\upgamma }{\text{e}}^{ - }_{{({\text{aq}})}} +^{ \cdot } {\text{OH }} + {\text{ H}}_{{2}} {\text{O}}_{{2}} +^{ \cdot } {\text{H }} + {\text{ H}}_{{2}} + {\text{HO}}_{{2}} /{\text{O}}_{{2}}^{ - }$$

R2 $${\text{e}}_{{({\text{aq}})}}^{ - } + {\text{ NO}}_{{3}}^{ - } \to {\text{NO}}_{{3}}^{{{2} - }}$$

R3 $$^{ \cdot } {\text{H}} + {\text{ NO}}_{3}^{ - } \to {\text{HNO}}_{3}^{ - }$$

### Surface morphology and textural properties

**Figure 2:**
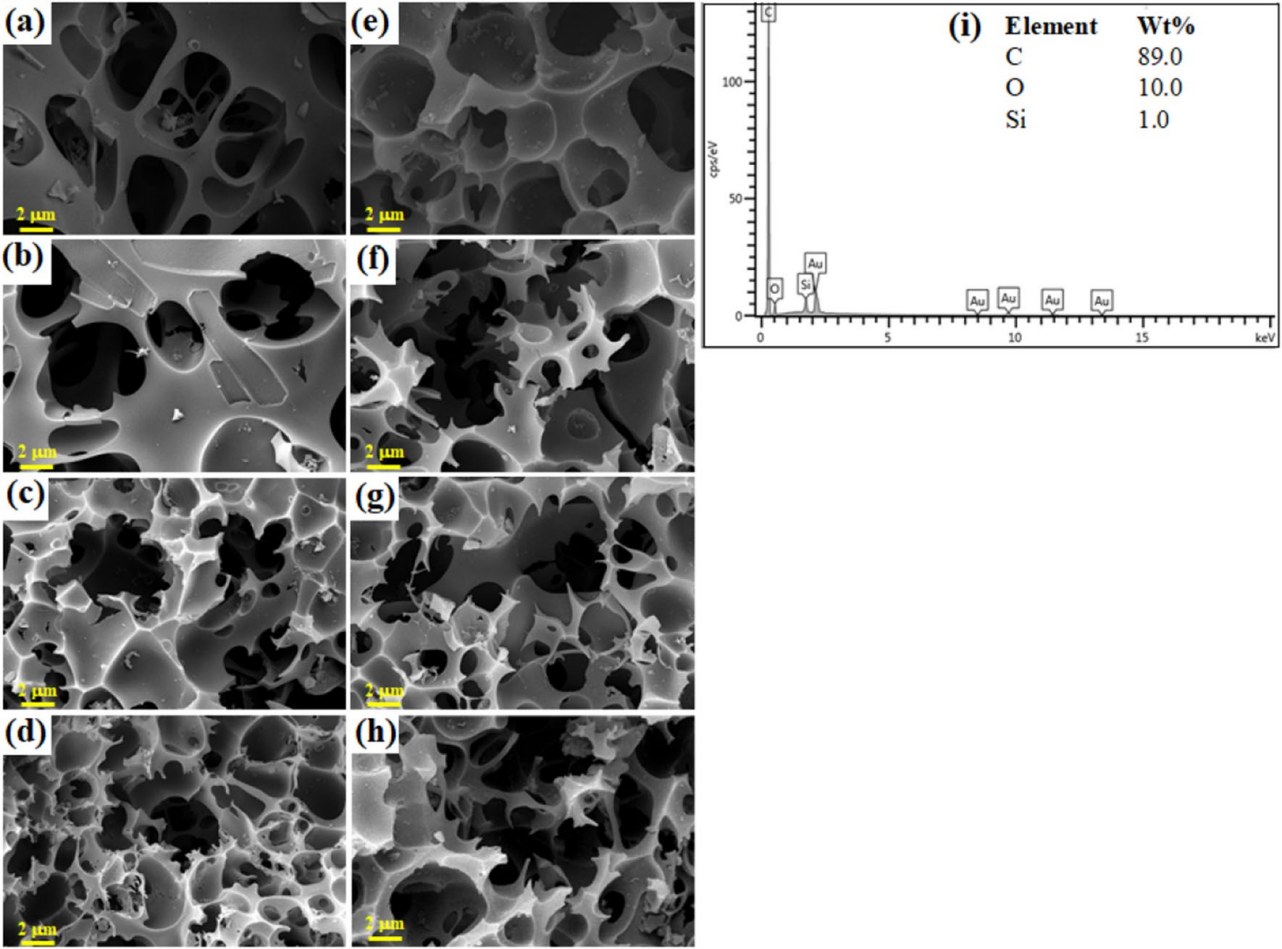
FESEM images of the synthesized ACs at 10.00 kV: (**a**) PP700_0 (**b**) PP700_25, (**c**) PP700_50, (**d**) PP700_100, (**e**) PP800_0 (**f**) PP800_25 (**g**) PP800_50 and (**h**) PP800_100, and (**i**) EDS spectrum of PP800_0.

FESEM images together with EDS analysis results, shown in Fig. 2, revealed the highly porous structure of the prepared materials with chemical composition containing around 89.0% carbon, 10% oxygen and 1% of silicon that may have contaminated from the cultivating environment (Fig. 2i). It should be emphasized that light elements such as hydrogen cannot be detected by the EDS elemental analysis. In addition, Au signals were observed in the EDS spectrum since gold was used in the conductive coating procedure for the detection. According to Fig. 2, the un-irradiated materials, PP700_0 (Fig. 2a) and PP800_0 (Fig. 2e) showed a porous structure with holes and cracks. As the material received gamma radiation at the total dose of 25 kGy, unnoticeable effect was seen in the case of PP700_25 (Fig. 2b). However, in the case of PP800_25 (Fig. 2f), it was clearly observed that some of the wall were broken forming interconnected networks of porous structures. These results may indicate that the consequent radiation effect can be governed dramatically by the activating temperature. When the total dose increased from 25 to 100 kGy, the interconnection between the holes obviously increased in both 700 and 800 °C sets (Figs. 2c,d,g,h). It is worth noting that at the maximum radiation dose of 100 kGy in the case of 700 °C, formation of small holes was noticed in addition. This was the evidence showing that small holes were created by bombardment of the carbon surface with the radiolytic oxidizing species before interconnecting of the small holes to form the networks. These highly porous structures are beneficial for supercapacitor performance as an increase in specific surface area can assist ions accessibility, charge storage and charge transfer in the active AC electrodes^8,37^.

**Figure 3:**
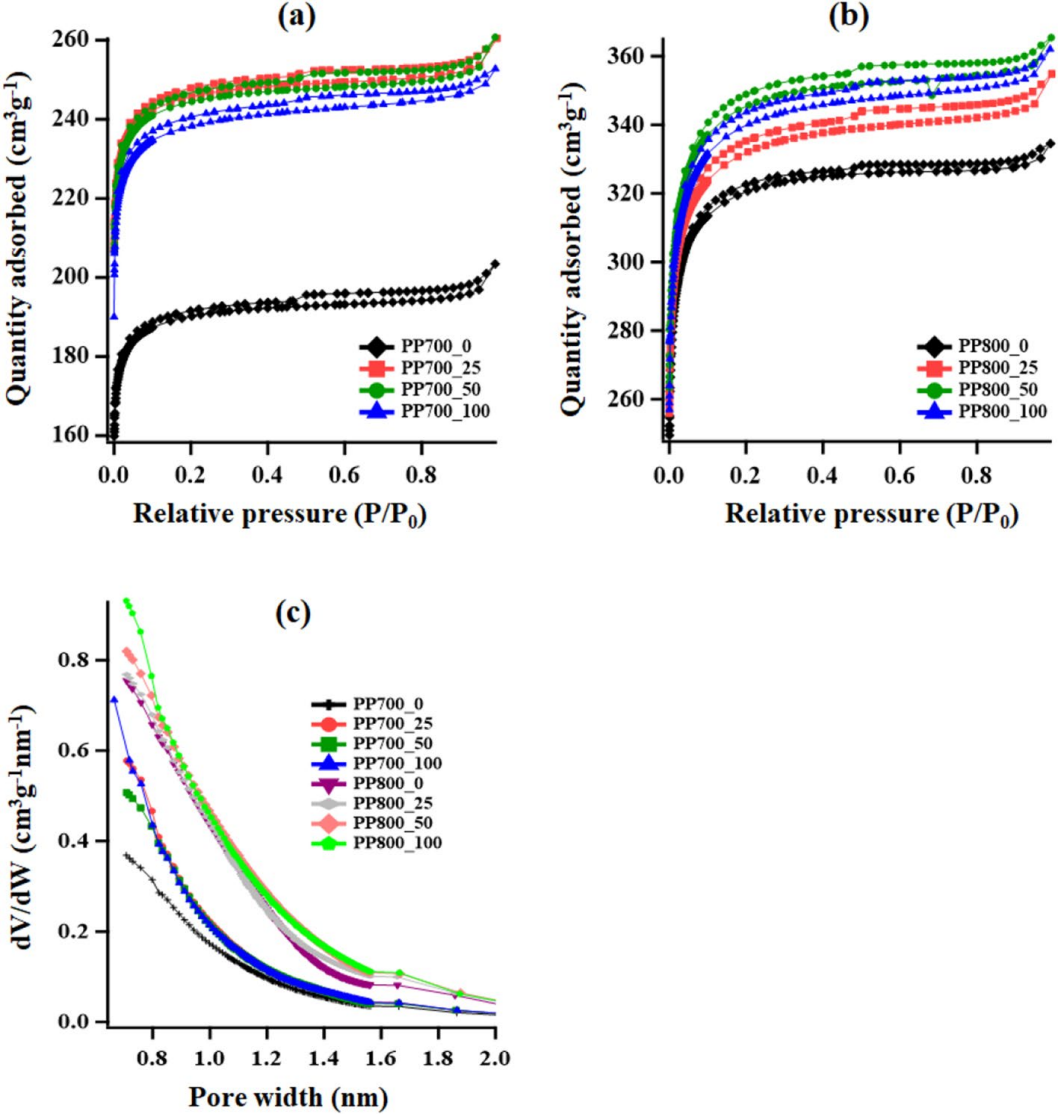
(**a**) N_2_ adsorption/desorption isotherms of PP700 series, (**b**) N_2_ adsorption/desorption isotherms of PP800 series and (**c**) pore size distribution of the synthesized ACs based on DFT model with Non-negative Regularization method.

More details about porosity were investigated by N_2_ adsorption/desorption isotherms with BET surface area analysis, t-plot and density functional theory (DFT) methods. From the N_2_ adsorption/desorption isotherms shown in Fig. 3a,b, the synthesized materials were classified as Type I and Type IV isotherms with Type H4 hysteresis according to IUPAC criteria^38,39^. For Type I, a steep N_2_ uptake was observed at low relative pressure (P/P_0_) indicating the presence of adsorbent-adsorptive interactions in very narrow pores (supermicropores, 0.7–2 nm) in the materials^40^. For Type IV, capillary condensation was accompanied by Type H4 hysteresis, revealing a mesopore characteristic. The data obtained therefor suggested the coexistence of micropores and mesopores in the graphitic carbons. The adsorbed quantities of the materials in the PP800 series were significantly higher than those in the PP700 series implying higher specific surface areas. The calculated porosity parameters: specific surface area, pore volume and average pore size are shown in Table 1. The total pore and micropore volumes were calculated by the t-plot method. The specific surface areas of the PP700 series lied between 770 and 996 m^2^ g^−1^ which were apparently lower than those in the PP800 series in the range of 1275–1365 m^2^ g^-1^. In both PP700 and PP800 series, after gamma irradiation the specific surface areas were clearly increased with the highest values of 996 m^2^ g^−1^ for the PP700 series (25 kGy) and 1365 m^2^ g^−1^ for the PP800 series (50 kGy). The pore sizes of the materials were found to fall in the micropore region with the average size around 1.6–1.7 nm. The pore size distribution, calculated using MicroActive for ASAP 2460 Version 2.01 from Micromeritics based on DFT model with a non-negative regularization method, can be seen in Fig. 3c. It is crystal clear that gamma irradiation with a relatively low radiation dose helps increase the specific surface area of the materials. In general, radiation processing can be used to improve material properties such as functional groups and porosity, and the quality of the desired properties normally increases with radiation dose before radiation damage occurs. In other words, at a lower radiation dose, a number of reactive species react with the material surface causing bond breaking and functional group alteration, and as a consequence, small pores are generated. At a high radiation dose, however a number of pores with larger dimensions are formed, resulting in the reduction of specific surface area. Saha et al. demonstrated that porosity of poly-[ethylene oxide] (PEO) powder was increased at gamma irradiation dose up to 3 kGy, and the porosity was decreased linearly when the dose was raised higher from 3 to 30 kGy^41^. This similar trend of radiation effect on materials was also reported previously by other research groups^42–44^. Therefore, the appropriate total dose used for a specific purpose of material development or improvement needs to be explored.

**Table 1:** Porosity parameters of the synthesized ACs.

Sample	Radiation dose (kGy)	S_BET_ (m^2^ g^-1^)	S_Micro_ (m^2^ g^-1^)	V_Total_ (cm^3^ g^-1^)	V_Micro_ (cm^3^ g^-1^)	Pore size_Avg_ (nm)
PP700_0	0	767	653	0.255	0.247	1.63
PP700_25	25	996	826	0.326	0.313	1.61
PP700_50	50	991	417	0.326	0.198	1.63
PP700_100	100	963	783	0.313	0.298	1.62
PP800_0	0	1275	887	0.416	0.346	1.62
PP800_25	25	1314	946	0.446	0.366	1.67
PP800_50	50	1365	912	0.460	0.358	1.66
PP800_100	100	1340	912	0.456	0.357	1.67

Note that the total pore and micropore volumes were determined using the t-plot method.

### Chemical properties

**Figure 4:**
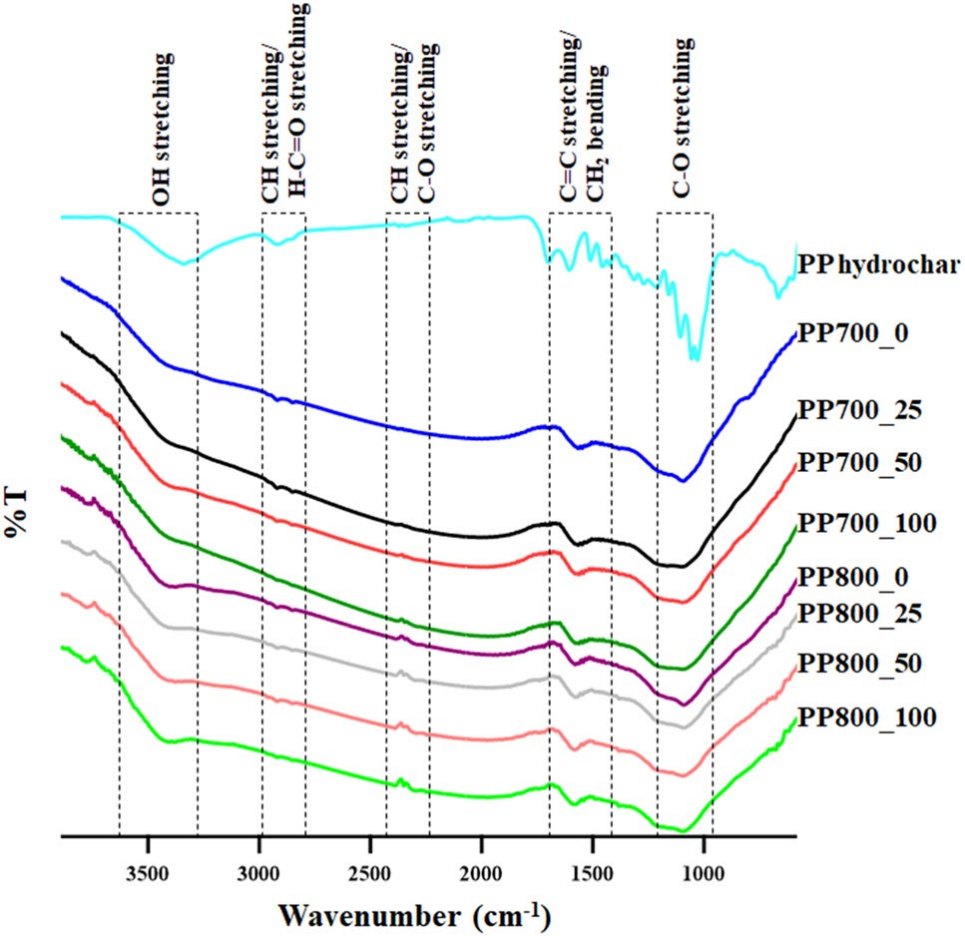
FTIR spectra of the hydrochar and synthesized ACs.

As it is known that hetero-atom containing functional groups on the material surface play an important role in its electrochemical properties. Especially in a pseudocapacitive electrode material, these functional groups help facilitate charge storage and charge transfer through rapid and reversible redox reactions at the electrode/electrolyte interface^45–47^. The surface functionalities of the materials were, therefore, investigated using FTIR. The results are shown in Fig. 4. As seen in Fig. 4, the hydrochar sample exhibited a broad peak around wavenumber 3340 cm^−1^ which belonged to OH stretching of carboxylic acid, alcohol and phenol. The peaks with small intensities at 2920 cm^−1^ and 2850 cm^−1^ were assigned to C–H stretching of methyl group and H–C=O stretching of aldehyde, respectively. Weak absorptions at 1700 cm^−1^ and 1600 cm^−1^ were due to vibrations of C=O band of aliphatic ketone/carboxylic acid and aromatic C=C^48^. The peaks around 1110 cm^−1^ and 1030 cm^−1^ were attributed to C–O stretching of ether and R–OH of primary alcohol. Therefore, the possible oxygen-containing groups in the hydrochar produced by H_2_SO_4_ hydrothermal carbonization were carboxylic acid, alcohol, phenol, aldehyde and ether. When biomass is hydro-thermalized, its structure is rearranged through reactions with water molecules. In a presence of an acid, the process is catalyzed leading to a faster decomposition of the bio-macromolecules^49^. The hydrochar formed by this technique was proven to have high surface area^50^ which in turn could well serve as a pre-treated material for activated carbon production. For the resultant ACs, the obtained spectra were similar to the hydrochar spectrum. However, the peak intensity of C–H around 2920 cm^−1^ clearly decreased and the C=O peak at 1700 cm^−1^ was unnoticeable after conversion into ACs. This was owing to evaporation of volatile species at high temperature during pyrolysis. The peak of C-O stretching around 2350 cm^-1^ was also observed in some samples. This may be due to contamination of CO_2_ during the measurement. The un-irradiated and irradiated ACs showed almost the same main functional groups. According to the FTIR spectra, the AC surface structures consisted of OH stretching (3420 cm^−1^), C–O stretching (2350 cm^−1^), CH stretching (2330 cm^−1^), C=C stretching (1580 cm^−1^), and C–O stretching (1110 cm^−1^) peaks which could be assigned to alcohol, phenol, carboxylic acid, aromatic ring and alkene. The peak assignments are summarized in Table 2^51^.

**Table 2:** The assigned functional groups from the FTIR spectra of the hydrochar and synthesized ACs^51^.

Wavenumber (cm^-1^)	Assigned functional group
3420–3340	OH stretching (alcohol, phenol, carboxylic acid)
2920	C–H stretching (methyl group)
2850	H–C=O stretching (aldehyde)
2350	C–O stretching
2330	CH stretching
1700	C=O stretching (aliphatic ketone, carboxylic acid )
1600	C=C (aromatic)
1580	C=C stretching (aromatic ring, alkene)
1110	C–O stretching (primary alcohol)
1030	R–OH

**Figure 5:**
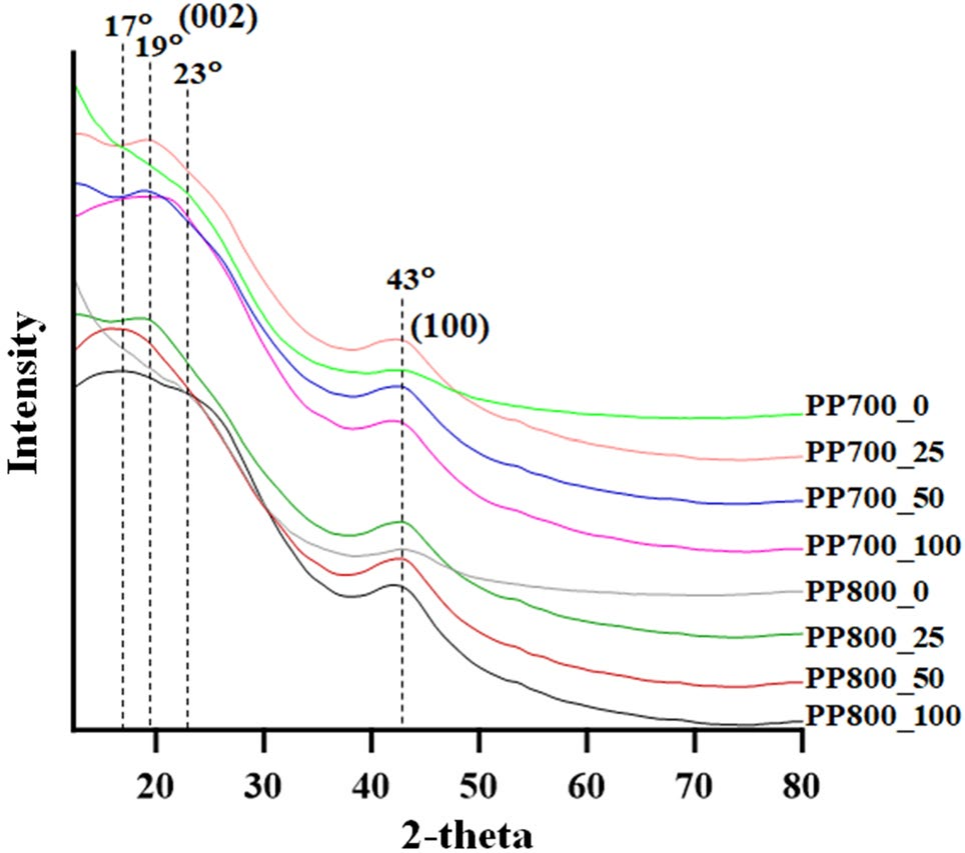
XRD patterns (Cu Kα radiation, λ = 1.54056 Å) of the synthesized ACs.

The crystallinity of the materials was studied using powder X-ray diffraction (XRD). The XRD patterns of the synthesized ACs are shown in Fig. 5. All samples exhibited two broad peaks, one in the range of 17–23° and another around 43° which were corresponding to 002 and 100 diffraction planes, respectively. These results revealed the amorphous and turbostratic structure of the porous materials that was microcrystalline with randomly orientation. The lower shift of 002 plane from 26° in pure graphite^52^ to 17–23° indicated the defective nature of the materials with a presence of a short range order of the graphene layers. For the un-irradiated ACs (PP700_0 and PP800_0), the diffraction peaks for 002 plane centered around 2-theta 23°. The peak, however, shifted to 20° in PP700_25, PP700_50, PP700_100 and PP800_25. In PP800_50 and PP800_100, the 002-plane peaks even lower shifted to 17°. This clearly suggests that the activation (pyrolysis) temperature and radiation dose play a significant role on the interlayer distance of the graphitic structure. Moreover, in both series the peak broadening in the XRD patterns of the samples was found to decrease after the materials were irradiated at all doses. As peak broadening indicates low level of crystallinity in the materials, therefore it can be mentioned that gamma irradiation at appropriate dose helps improve structural order in the carbon samples. With excess radiation energy, enormous structural changes may occur causing a decrease in the level of crystallinity. Although high pyrolysis temperature and high radiation dose may lead to low crystallographic order in a carbon material, its capacitive property may still remain with the compensating porosity.

**Figure 6:**
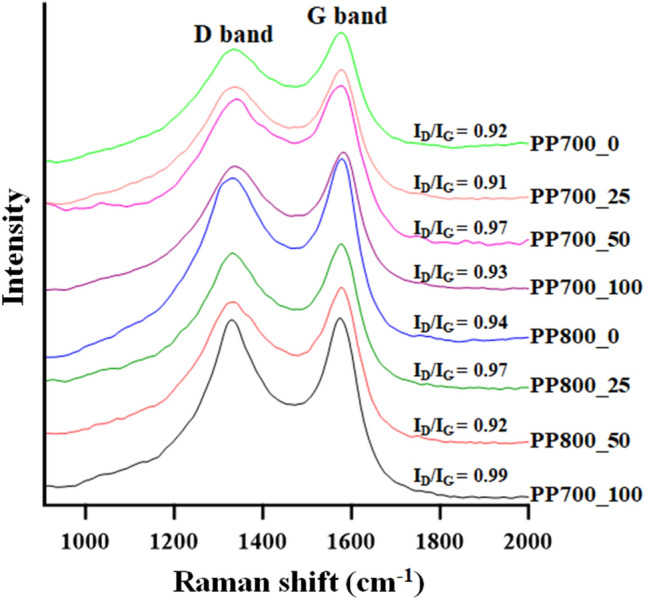
Raman spectra of the ACs obtained at laser wavelength 532 nm.

As it has been intensively demonstrated that degree of graphitization in carbon materials can effectively facilitate the charge and electrolytic ion transfer in supercapacitor application^53–56^, Raman spectroscopy was carried out to further study the graphitization degree of the prepared materials. The obtained results are shown in Fig. 6. The Raman spectra contained two board peaks around 1330 cm^−1^ (D band) which was associated to A_1g_ breathing mode of disordered sp^3^ carbon, and around 1580 cm^−1^ (G band) corresponding to E_2g_ in-plane vibration of sp^2^ carbon of conjugated chains and aromatic rings in graphitic layer^57^. It is well-established that the graphitization degree of the materials can be determined by the intensity ratio of D band over G band (I_D_/I_G_) based on Tuinstra and Koenig law, and the lower ratio value represents the higher degree of graphitization^58–60^. The values calculated for all samples as shown in Fig. 6 were in the range of 0.91–0.99, revealing amorphous nature of the materials with strong graphitization. The lowest I_D_-to-I_G_ ratios of both series, referring to the highest degree of graphitization or the highest ordered nature, were given by PP700_25 and PP800_50 with the values of 0.91 and 0.92, respectively. The high graphitization degree of PP700_25 was in good agreement with the XRD results indicating enhancement of ordered graphitic layers in the carbon material after irradiation. However, when the radiation dose was raised further to 50 and 100 kGy, interconnection between the micropores increased resulting in more disordered structures with lower graphitic degrees. For PP800 series, given 25 kGy of radiation, this level of radiation was sufficient to perturb the carbon structure thus increasing the number of sp^3^ carbons, but not enough to enhance the sp^2^ hybridization in the material. As the radiation energy was increased to 50 kGy, the perturbation was enough to change the sp^3^ units into sp^2^ domains. With higher radiation dose at 100 kGy, however, excess radiation caused more wall breakings similar to the PP700 cases and lowered the graphitization degree. Improvement of graphitization degree (sp^2^ hybridization) directly enhances the electrical conductivity of the material, hence supporting its capacitive characteristic.

### Electrochemical measurement

**Figure 7:**
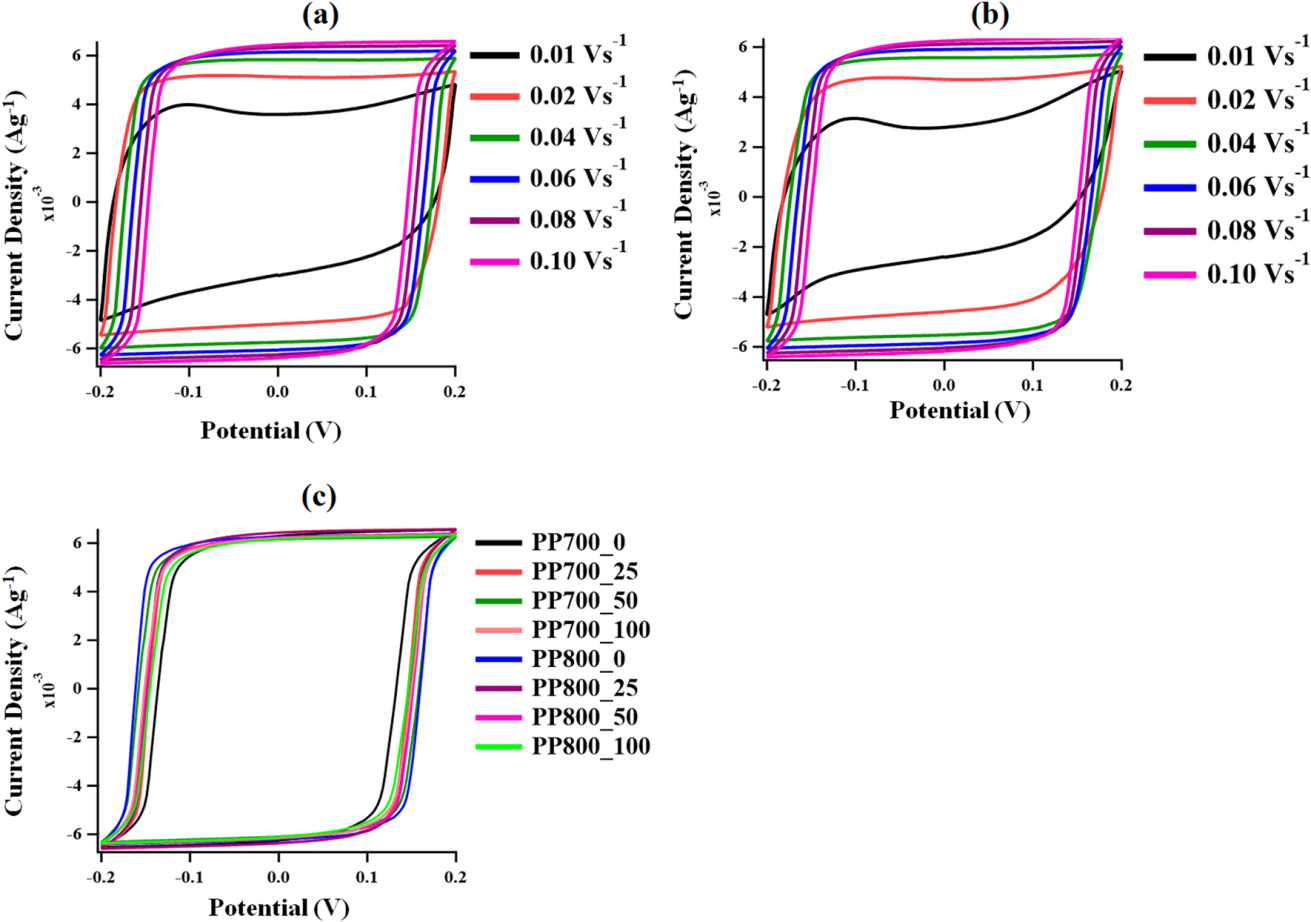
CV profiles obtained from a three-electrode system with Pt wire counter electrode and Ag/AgCl reference electrode in the potential window from − 0.2 to 0.2 V: (**a**) PP700_25 at varied scan rates, (**b**) PP800_50 at varied scan rates and (**c**) all synthesized ACs at 0.1 V s^−1^.

To get an insight into the electrochemical behaviors of the materials, supercapacitor electrodes were fabricated from the synthesized ACs and electrochemically tested in 1 M KOH electrolyte using a standard three-electrode system with Pt wire and Ag/AgCl as counter and reference electrodes, respectively. Cyclic voltammetry (CV) was used to detect the electrochemical behaviors of all prepared electrodes. The CV profiles at different scan rates (0.01–0.10 V s^−1^) in − 0.20 to 0.20 V potential windows of selected ACs from PP700 (PP700_25) and PP800 (PP800_50) series are demonstrated in Fig. 7a,b, respectively. According to Fig. 7, the voltammogram of each sample at a high scan rate revealed a near-perfect rectangular shape which was predominantly characteristic of EDLC. It was also found that the shapes of the current response remained unchanged when the scan rate increased from 0.02 to 0.10 V s^−1^. This suggests strong electrochemical reversibility that is a low barrier for electron transfer at the electrode/electrolyte interface^61^. This EDLC characteristic was attributed to the mesoporous nature of the carbonaceous materials which helped support the formation of the electric double layer^62^. Surprisingly at high scan rates pseudocapacitance behaviors of the samples, which was normally indicated by the presence of a redox peak at high potential, ascribed to the oxygenated functionalities on their surface was not detected. However, it was found that the curve recorded at the lowest scan rate (0.01 V/s) exhibited the least rectangular behavior. There are two possible explanations for this phenomenon. The first explanation is related to ion saturation at low scan rate. For an EDLC, initially, when the potential is increased, the ion concentration increases rapidly leading to increasing in current density. However, when ion concentration at the electrode surface reaches its maximum value, the electrode surface accumulation of ion gets slower, the current density decreases showing a CV curve with a hump^63^. The second explanation is related to the presence of redox reactions. The humps characteristic of redox reactions was attributed to the content of oxygenated surface functional groups as supported by the FTIR spectra. However, the recorded curve was asymmetric showing a broad reduction peak without a corresponding oxidation peak of equal magnitude, thus indicating some resistance. The similar results to our CV shapes at the lowest scan rate implying pseudocapacitive behavior were reported previously^64–67^. The explanation for this is that the Faradaic process can occur on both surface and interior of the electrode^68^, and such process is considered slow when compared to non-Faradic, such as EDLC. Therefore, it is possible that with a slow scan rate, more time allows redox reactions to occur at the interface and on the interior causing the deviation of the CV curve. On the other hand, at higher scan rates less time for redox reactions to occur; thus, the surface process dominates the energy storage mechanism, and the CV curves exhibit the ideal EDLC behavior. The influence of surface and bulk processes on CV scan rate for pseudocapacitive electrodes was studied earlier^69^. In this study, it was concluded that at a low CV scan rate, the mechanism was taken over by the bulk process. Therefore, the possible explanation for more deviation at the lowest scan rate could be related to ion saturation at the electron surface or redox mechanism. However, it is more likely that the deviation of the CV curve at the lowest scan rate was due to the presence of Faradaic process as supported by the FTIR and GCD results (discussed in the following paragraph). The mechanism of charge storage in the materials was, thus, a combination of EDLC and pseudocapacitance. The percentage of the capacitive contribution from each storage mechanism is particularly important. The study of EDLC and pseudocapacitance contribution under varied preparation conditions could be another research topic on its own, and we suggest this as future study. The voltammograms for all prepared AC electrodes are demonstrated in Fig. 7c.

**Figure 8:**
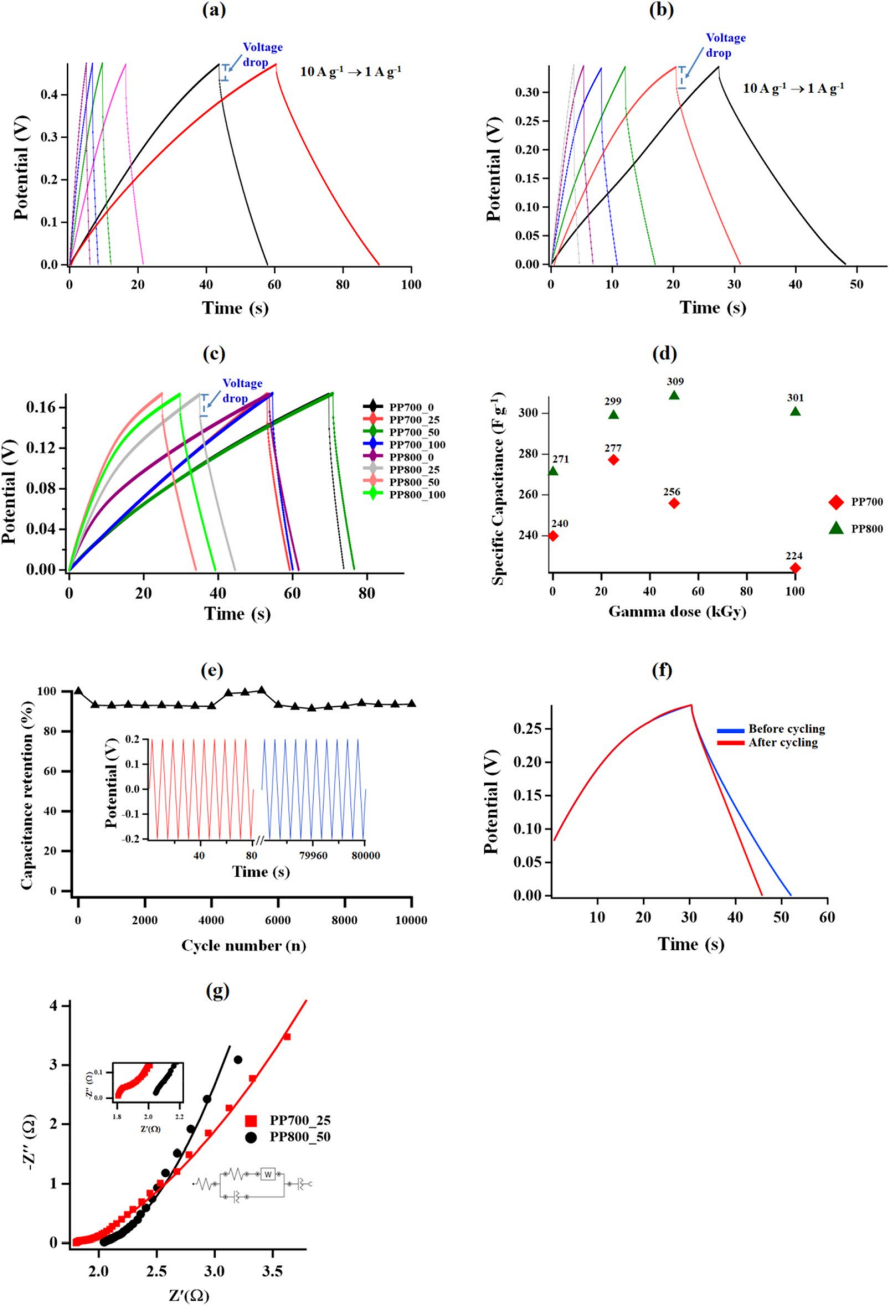
(**a**) GCD curves of PP700_25 at current densities 1–10 A g^−1^, (**b**) GCD curves of PP800_50 at current densities 1–10 A g^−1^, (**c**) GCD curves of all synthesized ACs at current density 1 A g^−1^, (**d**) specific capacitance (*C*_*p*_) from all ACs plotted against gamma dose, (**e**) capacitance retention (%) obtained from the PP800_50 electrode (inset depicts some of the initial and final charge discharge cycles), (**f**) GCD curves before (1st cycle) and after (10,000 cycles) cycling and (**g**) Nyquist plot of PP700_25 and PP800_50 electrodes measured in the frequency range of 10 kHz–0.1 Hz fit with the equivalent circuit.

Since it directly describes capacitive behavior, the current density is a crucial factor for supercapacitors. The capacitive behavior of the AC-derived supercapacitor electrodes was further investigated using galvanostatic charge–discharge (GCD) technique. The GCD curves from PP700_25 and PP800_50 at different current densities were illustrated in Fig. 8a,b, respectively. Similar results were obtained from both materials showing a deviation from a typical triangular shape at low current densities due to asymmetric charge and discharge durations. At lower current densities, the materials exhibit lower Coulombic efficiency which is defined as $\frac{discharging\, time}{{charging\, time}} \times 100\%$. In other words, the electrodes give high Coulombic efficiency at high current density reflecting their great storage ability which is a desirable feature of a supercapacitor material^70^. Furthermore, the deviations from symmetry in the GCD curves were due to the presence of the Faradaic charge transfer in additional to the EDLC mechanism. The Faradaic processes were possible with the presence of oxygen-containing surface functional groups indicated by the FTIR spectra and CV curves at the lowest scan rate.

Moreover, as it is well-established that specific surface area is the key factor governing the specific capacitance (*C*_*p*_) of a supercapacitor electrode, given the relatively high specific surface areas (767–1365 m^2^ g^-1^) discussed earlier, the prepared graphitic carbons were expected to provide high *C*_*p*_ values. The GCD profiles of all electrodes at current density 1 A g^−1^ were recorded (Fig. 8c) and used for *C*_*p*_ calculation according to Expression 1 (E1)^71^,

E1 $${C}_{p}=\frac{{I}_{m}\Delta t}{\Delta V}$$

where *C*_*p*_ is the specific capacitance (F g^−1^), *I*_*m*_ is the current density (A g^−1^), derived from ${I}_{m}=\frac{\mathrm{discharge current }(\mathrm{A})}{\mathrm{active mass on the electrode surface}}$, *ΔV* is the applied potential window and *Δt* is the discharging time. The specific capacitance and discharge time for the ACs were shown here as well at doses of 25, 50, and 100, and the controlled sample does not differ much. As the gamma-ray dose is increased, the discharge time and capacitive capacity of the electrodes first increase and then decrease, which can also be attributed to the increase in active surface area and graphitization degree that is a result of radiation at different doses. The PP700 (PP700_25) and PP800 (PP800_50) series samples were found to have the longest discharge times. This finding is in line with the EIS and CV findings that the ideal dose can improve electrode performance, indicating that the use of inadequate and excessive dosage levels must be avoided while producing electrodes^72^. All electrodes showed a sharp reduction in the initial voltage of the discharge curve in Fig. 8c, and this dip was caused by the electrolyte ions' diffusion-limited mobility within the electrode pores. The ESR (equivalent series resistance) of the supercapacitor cells is related to this restriction^73^. The calculated *C*_*p*_ values plotted against gamma radiation dose are shown in Fig. 8d. The values obtained are in the range of 224–309 F g^−1^. It was found that upon irradiation the *C*_*p*_ increased and after reaching the maximum value the number gradually decreased with radiation dose. These results could be well explained by the fact that irradiation at an appropriate dose led to enhancement of specific surface area, crystallinity and graphitization level in the carbonaceous structure; however, when the radiation dose was raised beyond this point, radiation damage occurred causing the decrease in all parameters, which were clearly proven by the results from FESEM, BET surface area analysis, XRD and Raman spectroscopy. The highest *C*_*p*_ values of each series were given by PP700_25 and PP800_50 with the values of 277 and 309 F g^−1^, respectively. Both samples with the highest *C*_*p*_ exhibited the highest specific surface area and graphitization degree within the series. Specific surface area is one of the most important parameters of a supercapacitor electrode as a high specific surface area can provide more adsorption sites for the charges and facilitate mass/charge transfer at the electrode/electrolyte interface. In turn, the amount of energy stored in the device is directly proportional to the specific surface area based on the Helmholtz model^74^. In addition to the specific surface area, the degree of graphitization is the primary factor supporting electrical conductivity in the material, thus improving charge transfer in the system as well. Unfortunately the two factors normally have a negative correlation, *i.e.*, in order to have a high specific surface area or porosity, the material needs to sacrifice its structural order or graphitization degree^53,75,76^. In the case of PP700_25 and PP800_50, the materials possessed high surface area and simultaneously retained great graphitization, hence having an excellent charge storage-charge transfer balance which ultimately resulted in the high *C*_*p*_ values. This is clear evidence proving that our preparation method is a promising choice for AC production with high surface area and high graphitization degree. More importantly, the *C*_*p*_ values obtained are considered high when compared with those reported in the literature. Table 3 shows the comparison of the *C*_*p*_ values from this work and those previously published based on similar chemical and physical activation methods with electrochemical measurements in a three-electrode setup. The electrode materials in all studies were biomass-derived activated carbons. The capacitance value of 309 F g^−1^ (from 1 M KOH electrolyte system) is comparable to the reported values, and it is considered relatively high. According to Table 3, 309 F g^−1^ is higher than the values from all similar studies with 1 M KOH electrolyte. This value is even higher than most of the specific capacitances obtained from a 6 M KOH electrolyte system. When a higher concentration of KOH electrolyte applied (in this case 6 times higher), the ions participating in the redox mechanism are increased; therefore, a higher specific capacitance should be expected, especially if the energy storage mechanism gets involved with pseudocapacitance. According to the comparison, it is obvious that a combination of chemical-physical activation and gamma irradiation in this work can be an effective method of AC synthesis for a high-performance supercapacitor electrode.

**Table 3:** Comparison of specific capacitance (*C*_*p*_) values at current density 1 A g^−1^ from this work and the literature based on chemical and physical activation methods with electrochemical measurement in a three-electrode setup.

Biomass	Chemical activating agent	Physical activating temperature (°C)	Electrolyte	*C*_*p*_ (F g^-1^)	References
Borassus flabellifer	H_3_PO_4_	900	1 M KOH	238.2	^77^
Kapok flower	KOH	700	6 M KOH	286.8	^78^
Corn flour	H_3_PO_4_	800	6 M KOH	151.2	^79^
	KOH				
Eucalyptus- globulus seed	H_2_SO_4_	900	6 M KOH	150.0	^17^
	KOH				
Corn stalk core	KOH	700	3 M KOH	140.0	^80^
Rubber seed shell	KOH	800	1 M KOH	123.0	^13^
Durian shell	KOH	800	1 M KOH	178.0	^13^
Palm petiole	KOH	800	1 M KOH	177.0	^13^
Castor shell	KOH	800	6 M KOH	481.0	^81^
Corn stalks	K_2_CO_3_	600	6 M KOH	203.5	^82^
Palm petiole	H_2_SO_4_	700	1 M KOH	277.0	This work
	KOH				
Palm petiole	H_2_SO_4_	800	1 M KOH	309.0	This work
	KOH				

A stability test with PP800_50 was conducted up to 10,000 cycles in a potential window of − 0.2 to 0.2 V. To obtain the capacitance retention, the *C*_*p*_ value at 1 A g^−1^ current density was calculated for every 500 cycles and compared with the first-cycle value. The results obtained are illustrated in Fig. 8e. In the first 4000 cycles, the specific capacitance (*C*_*p*_) was found to decrease to 92.6% of the initial value. This may be attributed to less migration of the ions through the porous structure. However, after 4500 cycles the value gradually increased and reached 100.4% at cycle 5500 indicating rearrangement of the electrode surface structure with more cycling numbers to promote faster ion migration. The capacitance retention slowly decreased and finally stabilized around 91.3–94.0% within 10,000 cycles ensuring the great endurance of the prepared AC-based electrode. Comparison between the GCD curves before (first cycle) and after (10,000 cycles) cycling can be seen in Fig. 8f, showing only little change in the energy storage performance.

The charge transportation ability at the electrode/electrolyte interface was studied using electrochemical impedance spectroscopy (EIS) at the bias potential of 0.1 V in the frequency range of 100 kHz–0.1 Hz. The results from PP700_25 and PP800_50 giving the highest specific capacitance values in each series are described in the form of Nyquist plots (Fig. 8g). According to Fig. 8g, the Nyquist plots are composed of two segments: a semicircle part in the high-frequency region and an inclined line in the low-frequency region. The semicircle part demonstrates the combination of electrolyte resistance and charge transfer resistance, whereas the linear segment represents the ionic diffusion process. Fitting the Nyquist plots using NOVA 1.11 software gave the equivalent circuit illustrated in Fig. 8g. According to the equivalent circuit, *R*_*s*_ is the uncompensated (internal) resistance of the cell, *R*_*p*_ is referred to the charge transfer resistance, *CPE* is the Constant Phase Element and *W* is the Warburg impedance showing the ionic diffusion through the diffusion zone. The *R*_*s*_ values obtained for PP700_25 and PP800_50 electrodes were 1.8 and 2.0 Ω, respectively. The *R*_*s*_ value was lower for the PP700_25, which suggests that introducing low amount of Gamma dose at 700 °C reduces the internal resistance of the electrodes. The values of *R*_*p*_ for both electrodes were around 70 mΩ. Considering the particularly small *R*_*p*_ values, the fabricated electrodes are suitable for supercapacitor application.

## Conclusion

In this study, activated carbons (ACs) were successfully synthesized from palm petiole using a three-step strategy composed of H_2_SO_4_ hydrothermal carbonization, KOH-activating pyrolysis and gamma radiolysis under oxidizing condition. The prepared graphitic carbons were highly microporous with porosity greatly influenced by activation temperature and gamma dose. Given higher activation temperature, a larger specific surface area was clearly developed. Gamma-induced modification of the carbon surface structure resulted from the bombardment of the carbon surface with radiolytic oxidizing species creating interconnection between the micropores. The appropriate radiation dose needed to be explored as excess radiation energy caused larger pores leading to low specific surface area. The maximum specific surface area of 1365 m^2^ g^−1^ was given by the AC pyrolized at 800 °C with 50 kGy of gamma dose (PP800_50). The surface functional groups of the porous carbons could be assigned to alkene, aromatic ring, alcohol, phenol and carboxylic acid. The level of crystallinity was increased after irradiation with pronounced (002) and (100) planes. The activation temperature and radiation dose played a significant role on the range order and the interlayer distance of the graphitic structure. Excess radiation caused more wall breakings, thus lowering the graphitization degree. When an optimized radiation dose was applied, however, high surface area and great graphitization (high conductivity) can be both obtained. The produced supercapacitor electrodes showed outstanding electrochemical properties with a dominant EDLC mechanism. The specific capacitance of PP-AC electrodes was apparently dictated by the specific surface area. The PP800_50 electrode was found to be the most suitable for supercapacitor application. It showed the highest specific capacitance of 309 F g^−1^ and exhibited small total internal resistance and polarization resistance around 2.0 and 70 mΩ, respectively, with excellent endurance up to 10,000 charge–discharge cycles. Therefore, the present research successfully demonstrated an efficient and environmentally friendly approach of biomass-derived activated carbon synthesis for supercapacitor electrode application.

## Data Availability

The data that support the findings of this study are available from the corresponding author upon reasonable request.
